# The bane of publishing a research article in international journals by African researchers, the peer-review process and the contentious issue of predatory journals: a commentary

**DOI:** 10.11604/pamj.2019.32.119.18351

**Published:** 2019-03-14

**Authors:** Elvis Enowbeyang Tarkang, Luchuo Engelbert Bain

**Affiliations:** 1 Department of Population and Behavioural Science, School of Public Health, University of Health and Allied Sciences PMB 31 Ho, Ghana; 2 HIV/AIDS Prevention Research Network Cameroon, Kumba, Cameroon; 3 The Pan African Medical Journal, Nairobi, Kenya; 4 Athena Institute for Research on Innovation and Communication in Health and Life Sciences, Vrije University, Amsterdam, Netherlands

**Keywords:** Peer-review, African journals, predatory journals, international journals, African academics and researchers

## Abstract

Scholarly publication in a peer-reviewed journal is the highest form of disseminating research findings. However, the process of publishing in peer-reviewed journals remains a daunting task for researchers and academics in Africa. This commentary will assist authors in Africa to understand the peer-review process, to appreciate the length of time it takes for a manuscript to be published and to encourage them to publish in local peer-review journals. The authors argue that the peer-review process is essential because it acts as a quality control mechanism to ensure that valid and reliable research is published. Although peer review does not guarantee exclusive publication of reliable and valid research, it remains central in the scientific activity. Authors need to take the comments from reviewers and editors, even in cases of rejection seriously and in the positive sense, inorder to improve upon the quality of their work. Rejections by some journals happen not to be scientifically grounded. It happens that African authors suffer more from this flaw. This could justify why some naïve authors easily turn to publish in predatory journals. The authors argue that publishing in local journals is imperative for Africans scholars. Therefore, initiatives to encourage publication in these journals are highly needed.

## Commentary

Scholarly publications remain the main vehicles for disseminating research findings, with research publication in peer-reviewed journals being at the peak of dissemination. Academic activities that contribute to the advancement of knowledge, must be published in sufficient details to enable other potential researchers to replicate the study [1]. However, the process of publishing in peer-reviewed journals remains a daunting task especially for researchers in Africa. There are several reasons why researchers and academics in Africa are under tremendous pressure to publish, including contract renewals, advancement and promotion criteria, academic success, fame amongst others [1]. The International Committee of Medical Journal Editors (ICMJE) defines peer-review as the critical assessment of manuscripts submitted to journals by experts who are not part of the editorial staff of the journals [2]. The current article will assist authors in Africa to understand the peer-review process and its importance, to appreciate the length of time it takes for a manuscript to be published after submission and to encourage them to publish in local peer-reviewed journals.

**The importance of peer-review:** Peer-review is the most important part of the scholarly publication process. It acts as the quality control mechanism put in place by journals, with the aim of selecting quality articles for publication, by filtering out studies that have been poorly conceived, designed and executed, with the selection criteria based upon the scientific merit and validity of the article. Peer-review also aims to improve the quality of the manuscript before publication and to check against plagiarism and unethical considerations. Therefore, the main functions of the peer-review process are to help maintain standards and ensure that the reporting of research work is as empirical and accurate as possible [3].

**The peer-review process:** The peer-review process is similar for most journals, howbeit with minor variations among journals. The first step of the process is the editorial triage; once the corresponding author submits a manuscript either as an email attachment or through the online submission process it is logged in and checked to make sure that the submission is complete and has been prepared according to the journal's scope, instructions to authors and submission guidelines [4]. At this time an acknowledgement receipt is sent to the author(s) to let them know that their manuscript has been received. Each manuscript is then read by an editor to assess its suitability for the journal according to the journal's guidelines and scope and to make sure the manuscript does not contain an unacceptable level of plagiarism. At this point, a manuscript could be rejected without additional review if it does not meet the conditions stated above, and the author(s) notified [5]. If a manuscript is not rejected when first received, it is then sent out by the chief editor of the journal for review to a minimum of two reviewers who are part of the journal's cadre of reviewers. The review could be single-blinded or double-blinded. In the single-blinded review, the reviewers' identities are withheld from the authors but the reviewers are aware of the authors' identities [6]. This type has the potential for bias because works originating from certain authors, institutions, or geographic locations may have the potential to be treated more or less critically. In the double-blinded review, the identities of the authors are also masked during the review process. Both the authors and the reviewers are unaware of each other's identity. This type of review is the type employed by most journals [4], though with some limitations; manuscripts that cite most of the submitting authors' previous research may be difficult to mask effectively [6]. However, since the reviewers are experts in their various fields, it is assumed that they are on the side of science and a thorough review without bias would be done notwithstanding the identities of the authors. A good review fills the gaps, improves the manuscript and stretches the authors. It consists of constructive criticism and occasionally praises. Sometimes it may happen that a reviewer is not knowledgeable enough in the field or he/she is a competitor. This is filtered at the editorial level by inviting multiple reviewers.

Once reviewers are chosen and they accept the assignment, the real process begins. Most reviewers are giving timelines and some form of a checklist that covers all the sections of the manuscript according to the journal guidelines [4]. However, this checklist more often applies to “original research papers”, with other types of submissions such as Short communications, Commentaries and Review articles having different criteria for assessment. Once the review process is complete within the stipulated timeframe, the reviewers return their comments and recommendations to the chief editor (via the online submission system or email), who assesses them collectively and then makes a decision on either to reject the manuscript (either outright or with encouragement to resubmit), to accept it pending major or minor revisions, or to accept it as it was submitted. For manuscripts accepted pending revision, the authors must submit the revised manuscript that will go through all or part of the submission process, with a rebuttal letter addressing all the reviewers' concerns [4]. Upon a satisfactory revision of a manuscript following the reviewers' suggestions, it will be accepted and put into the production process to be prepared for publication. The production process, controlled by a production editor, or publisher takes the article through a copy editing, typesetting, inclusion in a specific issue of the journal; then printing and online publication. Copy editing seeks to ensure that an article conforms to the journal's style, that all the referencing and labeling are correct; and that there are no spelling and grammatical mistakes. Typesetting deals with the appearance of the article-layout fonts and headings [4]. The process from submission of the manuscript to actual publication takes months to complete. So, authors from Africa must exercise some patients if they value quality in their published work.

**The bane of publishing in top-quality international peer-reviewed journals for African scientists:** Africa accounts for only 2% of the world research output. This is partly due to the fact that research papers from Africa are often rejected when submitted to international journals for publication [7]. Due to lack of sustainable local journals [7], African academics and researchers have no choice than to strive to publish in internationally renowned peer-reviewed journals, which are very competitive, in order to ensure academic promotion. This high competition makes academic publication in high-quality international journals, a mirage for African scientists and researchers.

### The reasons why academics in Africa find it difficult to publish in international peer-reviewed journals

***The high rejection rate for manuscripts from Africa:*** Research originating from Africa often address local problems and are meant for the local audience. The findings are of little interest to international journals; therefore, these manuscripts are often rejected when submitted to international journals for publication. It is possible that some editors think upfront that research, in some fields, is automatically of lower quality.

***Financial constraints:*** Journals may be either close-access or open-access; however, the peer-review process is the same. Most open-access journals attract publication fees, which are paid by the author(s) of the manuscript, while consumers of research access the journals free of charge. Meanwhile, with most close-access journals, accepted articles are published free of charge and the users pay some fee before they can access the articles. In a rapidly growing scientific world and in the internet age we find ourselves today, coupled with the poverty situation in Africa, most consumers of research go for open-access journals where they can access information free of charge even via their mobile electronic devices. This has left most scientists and researchers in Africa with no choice than to publish in open-access journals with the challenge of paying publication fees. The high financial cost of publishing in some international peer-reviewed open-access journals means that much of the research done in Africa remains invisible to the rest of the world because of the inability for most scientists in Africa to afford publication fees [8].

***Turnaround time for peer-review in top quality journals:*** Most accredited and high-impact factor journals that are indexed in databases like Medline, PubMed and Scopus have a very long turnaround time for the peer-review process to be completed, sometimes as long as two years. Sometimes before the paper is actually published, the information has become obsolete or redundant. It is against this backdrop that most African researchers and scientists prefer to publish their papers in low-impact-factor journals or in the so-called “predatory journals”.

***The “publish or perish” conundrum:*** The “publish or perish” conundrum has caused many researchers from Africa to publish their works in low-quality journals, often without rigorous peer-review process, because they are under tremendous pressure to publish in order to gain promotion especially in the academia where publication is often regarded as the sole currency for promotion.

### The reasons why researchers and academics in Africa feel obliged to publish in international journals

***The publishing abroad syndrome:*** Researchers and academics in Africa have been made to feel that they will gain worldwide recognition and reputation when their papers are published outside the continent. They have been brain-washed to feel that works published outside Africa are better than locally published ones [9].

***The high mortality rate for African journals:*** The high mortality rate of journals in Africa is another major reason why researchers in Africa would prefer to publish their works in international journals. Many of these journals do not even acknowledge receipt of papers sent to them. In some cases, a manuscript could be delayed for a considerable length of time, thereby making the information they provide obsolete or redundant when it is eventually published. Foreign journals always have the advantage of getting information abstracted, indexed and reviewed by major international abstracting and indexing services, thereby increasing the visibility of authors. This serves as an attraction to African researchers.

**Advantages of publishing in African Journals:** There are many reasons why researchers in Africa should patronize local journals: the impact factor and usability of these journals would be raised, thereby reducing their mortality rates; research findings from Africa that are often meant for the local audience because they address local issues, which are of little interest to international audience, would not be lost when the articles are being published in local African journals. Articles published in international journals are often inaccessible to the African audience because of the high cost of accessing these articles.

**The contentious issue of Predatory Journals:** “Predatory journals are ones that exploit the page fee model for self-gain. They transgress all the rules of research integrity and in most cases, they have no clear focus area” [10]. Due to the difficulties encountered by African researchers in getting their works published internationally, they patronize Predatory or standalone journals either knowingly or unknowingly. According to Beall (2015) [10], characteristics of Predatory Publishing include: Accepting articles quickly with little or no peer review or quality control; notifying academics of article fees only after papers are accepted; aggressively campaigning for academics to submit articles or serve on editorial boards; Listing academics as members of editorial boards without their permission; appointing fake academics to editorial boards; mimicking the name or website style of more established journals; misleading claims about the publishing operation, such as a false location; Improper use of ISSNs; Fake or non-existing impact factors; The publisher has an optional “fast-track” fee-based service for expedited peer-review which appears to provide assured publication with little or no vetting. Due diligence must therefore, be performed by researchers before submitting their manuscripts to a journal to ascertain that it provides full, verifiable contact information including address, on its site. We have to be cautious that a journal´s editorial board contains recognized experts with full affiliations. Some of the editorial board members could be contacted and asked about their experience with the journal. We must also make sure that the journal prominently displays its policy for author fees. Researchers should also be wary of e-mail invitations to submit to journals or to become editorial board members; read some of the journal's published articles and assess their quality; contact past authors to ask about their experiences; and check that a journal's peer-review process is clearly described and try to confirm that a claimed impact factor is correct [10]. Notwithstanding the fact that there are predatory journals and publishers, a single individual does not have the authority to determine if a journal is predatory or not and Beall's description of predatory journals favours mainstream international journals and is biased against open access journals from low and middle-income countries including those from Africa. So, this description should also be taken with caution by researchers and academics from Africa.

## Conclusion

The peer-review process helps refine and improve the quality of the published article by addressing the thoughtful comments raised by reviewers and editors. This can only happen when expert reviewers take time to participate in the peer-review process and evaluate submissions with care and sensitivity. The editors and reviewers of peer-reviewed journals are committed to utilising a stringent but fair review process in order to assist authors who submit scholarly work for publication [5]. However, there are some journals which claim to be peer-reviewed but publish any manuscript that is submitted within a very short time period, sometimes 24 hours, without going through the elaborate peer-review process as explained in this commentary. It is almost impossible for peer-review to be done and an editorial decision taken within 24 hours, not even one week after submission of a manuscript. African authors should shy away from such journals. This notwithstanding, if editors find a particular submission of interest to their journal, they may expedite the review process and a decision reached within one week. Most of the research work that is undertaken in Africa, are meant for the local audience and consumption. So, it makes much sense if such works are published in African journals for the African audience. It is incumbent on universities and research institutes in Africa, to make available to researchers and academics on a yearly basis, a list accredited peer-review journals from Africa, and to encourage them to publish their works in these journals. By doing this, the impact factor of these journals would be raised and they would become renown and could compete with other international peer-reviewed journals. Local content should be encouraged in academic publishing in Africa. African scientists should come up with an “African Committee of Journal Editors” who will have the mandate and authority to decide whether a journal is predatory or not, and also come out with a list of accredited African journals and encourage African Scientists and Academics to publish their works in such journals. The research and scientific world should collaborate with governments in Africa to support Local journal and publishing houses. This could go a long way to reduce the mortality rate of African Journals. Universities and research institutes in Africa should be encouraged to develop their own journals and publishing houses so as to create a direct avenue for academics and researchers to publish their research findings.

## Competing interests

The authors declare no competing interest.
